# Transcriptome analysis of bagging-treated red Chinese sand pear peels reveals light-responsive pathway functions in anthocyanin accumulation

**DOI:** 10.1038/s41598-017-00069-z

**Published:** 2017-03-03

**Authors:** Songling Bai, Yongwang Sun, Minjie Qian, Fengxia Yang, Junbei Ni, Ruiyan Tao, Lin Li, Qun Shu, Dong Zhang, Yuanwen Teng

**Affiliations:** 1 Department of Horticulture, Zhejiang University, Hangzhou, 310058 Zhejiang Province People’s Republic of China; 2 The Key Laboratory of Horticultural Plant Growth, Development and Quality Improvement, the Ministry of Agriculture of China, Hangzhou, 310058 Zhejiang Province People’s Republic of China; 3 Zhejiang Provincial Key Laboratory of Integrative Biology of Horticultural Plants, Hangzhou, 310058 Zhejiang Province People’s Republic of China; 4 Institute of Horticulture, Yunnan Academy of Agricultural Sciences, Kunming, 650205 Yunnan Province People’s Republic of China; 5 College of Horticulture, Northwest A & F University, Yangling, 712100 Shaanxi Province People’s Republic of China

## Abstract

Bagging is an efficient method to improve fruit colour development. This work reported a transcriptome analysis using bagging-treated red Chinese sand pear peels. In total, 8,870 differentially expressed genes were further analysed by a weighted gene co-expression network analysis and early-, middle- and late light-responsive genes were identified. An annotation analysis revealed several pathways involved in the different responsive stages. The presence of *LONG HYPOCOTLY 5*, *CRY-DASH* and a *CONSTANS-like* transcription factors among the early light-responsive genes indicated the pivotal role of light, especially blue light, in the biological changes that occurred after bag removal. Other light-responsive transcription factors were also identified from the three light-responsive stages. In addition, the light-responsive pattern of anthocyanin biosynthetic genes differed among the biosynthetic steps. Although yeast-one hybrid assay showed that most of the structural genes were regulated by PpMYB10, their different temporal expressive pattern suggested that besides PpMYB10, other light-responsive transcriptional factors were also involved in the regulation of anthocyanin biosynthesis. In summary, our transcriptome analysis provides knowledge of the transcriptional regulatory network operating during light responses, which results in anthocyanin accumulation and other significant physiological changes in red Chinese sand pear peels after bag removal.

## Introduction

Pear (*Pyrus*) is an important fruit in China. Traditional pears are usually green or yellow but in recent years, red pears have gained consumer preference because of their attractive appearance and nutritional value. To date, red pear resources (or sports) have been identified in all four cultivation groups in China. Because the colour developmental pattern differs among these groups^1^, they are good research materials for studying the regulatory mechanism of fruit colouration. The red colouration of red Chinese sand pears (RCSPs, *Pyrus pyrifolia*) mainly depends on the concentration and composition of anthocyanins in the fruit peel^1–3^. Anthocyanin is synthesised in the cytosol and transported to the vacuole by a glutathione S-transferase (GST)^4^. The biosynthesis of anthocyanin involves several well-characterised enzymes, including chalcone synthase (CHS), chalcone isomerase (CHI), flavanone 3-hydroxylase (F3H), flavonoid 3′-hydroxylase (F3′H), dihydroflavonol 4-reductase (DFR), anthocyanidin synthase (ANS) and UDP-glucose: flavonoid 3-O-glucosyltransferase (UFGT). The spatial and temporal expressions of the genes coding these enzymes are regulated at the transcriptional level by various transcription factors (TFs), especially the well-studied MBW complex, which is composed by MYB, basic helix-loop-helix (bHLH) and WD40^5, 6^.

In many species, R2R3 MYB TFs were characterized as important TFs for the activation of anthocyanin biosynthesis genes. In apple, MdMYB1/A/10 act as positive factors of anthocyanin synthesis in skin and flesh when co-expressed with MdbHLH3 or MdbHLH33^7–9^. In grapes, several R2R3 MYBs were isolated and implicated in anthocyanin biosynthesis^10^. In strawberry, two R2R3 MYBs are involved in anthocyanin biosynthesis. FaMYB10 acts as an activator, whereas FaMYB1 acts as a repressor. Interestingly, both the activator and the repressor showed high expression levels at the fruit ripening stage, suggesting that they may fine tune gene expression to balance the accumulation of anthocyanin^11–13^. Pear PpMYB10 (misassigned as PyMYB10 previously) contributes to the red pigmentation of RCSPs^14^.

In fruit crop cultivation, poor colouration has long been a problem owing to unfavourable environmental conditions and the varietal differences in adaptability. To improve fruit colouration, various management practices, such as leaf removal, fruit bagging, reflective films, evaporative cooling and postharvest artificial irradiation, have been developed^15–19^. In China, paper bagging during fruit development is used to enhance the red pigmentation of RCSP fruit skins^1, 2^, as well as to protect fruits from birds and insects, and alleviating fruit russeting. A commercial paper bag is usually composed of two light-blocking layers, while some have an additional translucent inner layer (usually red). The fruits are covered with these bags 1 month after full blossom and the bags are removed 10–15 days before the expected harvest date. This is referred to as the ‘bagging treatment’ in the current report.

The bagging treatment is basically a light re-exposure process, although the air composition, temperature and humidity around the fruit may also change, resulting in various physiological changes in the fruit. Fruit bagging efficiently improves the coloration of RCSPs. Fruit bagging works in the following two ways to improve fruit colouration: (1) The optimum light condition can be controlled by the timing of the bag removal, thereby achieving the desired fruit colour, which often occurs at maturity. (2) Bagging can increase the light sensitivity of the fruit, enhancing the development of an intense and uniform red colour^20^.

The mechanism of light-induced anthocyanin biosynthesis has been well characterized in model plant Arabidopsis as a part of photomorphogenesis. It requires the light-responsive elements including phytochromes and their downstream factors, such as CONSTITUTIVELY PHOTOMORPHOGENIC 1 (COP1), LONG HYPOCOTYL 5 (HY5) and *etc* ^21^. Based on these reports, several attempts have been carried out to explain the biological process of fruit coloration using high-throughput approaches^22–24^. The involvement of light-responsive genes in fruit coloration was confirmed but the detailed regulatory pathway may be different from that in model plants. For instance, Arabidopsis HY5 directly binds to the promoter of *AtMYB75* to activate its expression but there are no evidence showing the activating effect of apple HY5 on the expression of *MdMYB1* (unpublished data) although HY5 can bind to its upstream region^22^. Therefore, the mechanism for the light-induced anthocyanin biosynthesis in fruit peel needs further analysis.

In this study, we analysed the transcriptomes of RCSP fruit peel samples harvested at the initial (0 h)-bag removal stage, along with early (6 h), middle (24 h) and late (144 h) stages after bag removal. Bagged fruit sampled at the same times served as controls. By constructing co-expression networks, we identified the genes involved in early, middle and late light-responsive reactions and further analysed the differential regulation of the structural genes involved in anthocyanin biosynthesis. Our work identified sequentially expressed light-responsive genes, which will help us to further clarify the mechanism of bagging-treatment-induced colouration and enrich our knowledge regarding the regulation of anthocyanin biosynthesis. The data obtained from RNA sequencing (RNA-Seq) will also provide researchers with useful basic information for data mining and will facilitate further experiments on light-induced fruit colouration.

## Results

### Changes in fruit pigmentation patterns in the peel of RCSP ‘Meirensu’ fruit

**Figure 1:**
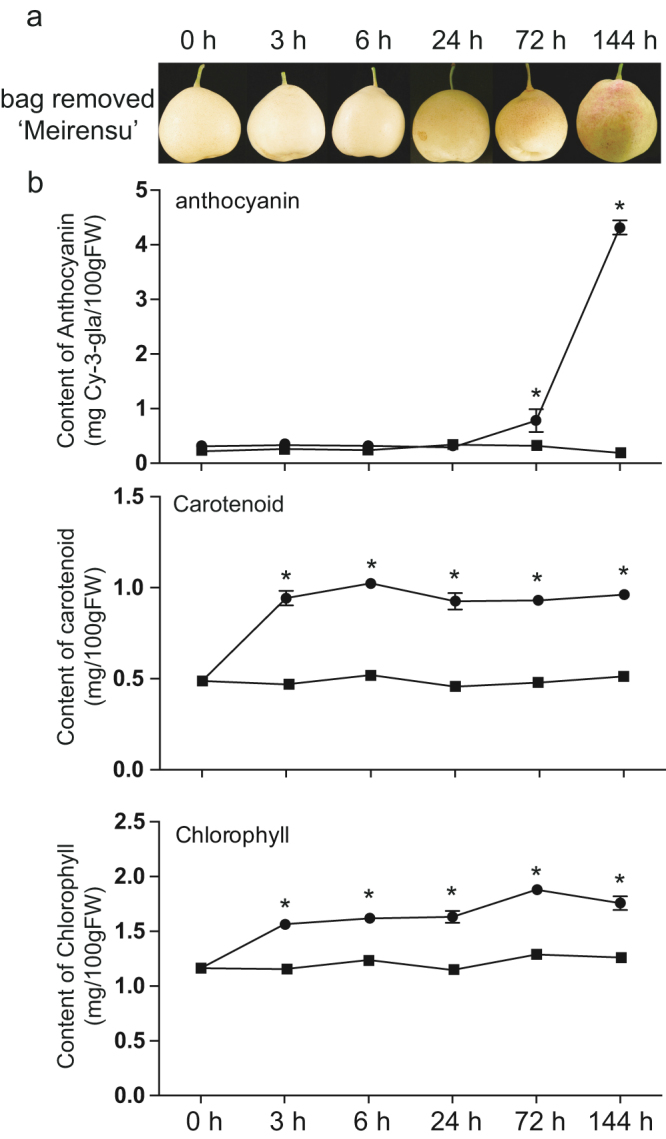
Changes in pigmentation of fruit peel of red Chinese sand pear in bagged and bag-removed samples. (**a**) Photos for bag-removed pear ‘Meirensu’. (**b**) Content changes of anthocyanin (up), carotenoid (middle) and chlorophyll (down) in fruit peel after bag removal. The labels of the samples were displayed as “hours after bag-removal” (h). The graphs showed the average values from three independently sampled fruits as biological replicates. Error bars are the standard deviation. Asterisk indicates ﻿*p﻿* < 0.05.

Paper bags were removed ten days before predicted harvest day. By visual inspection, the fruits removed the bags were light yellow for 24 h and gradually turned red after 72 h, while the control samples remained light yellow (Fig. 1a and data not shown). The red colouration was accompanied by an elevation in the anthocyanin content. In addition, the carotenoid and chlorophyll contents in the bagged fruit were very low and slightly increased immediately after bag removal, prior to the anthocyanin accumulation (Fig. 1b).

### Library construction and sequencing

The fruit peel sampled at 0 h, 6 h, 24 h and 144 h after bag-removal, and their corresponding bagged fruit peel (0H, 6H, 24H, 144H, 6HC, 24HC and 144HC) were subjected to total RNA extraction and an RNA-Seq analysis. High-throughput sequencing generated 20.37–24.11 million (M) 100-bp paired-end reads from each library (Table 1). After a stringent quality filtering process, 94.20 Gb of clean data (96.40% of the raw data) were obtained, with a Q30 percentage ≥80%. The counts of clean reads per library ranged from 19.71 to 23.86 M (Table 1). Reads were mapped to the reference genome sequence of Chinese white pear ‘Dangshansuli’ (*Pyrus pyrifolia* White pear group, misassigned to *Pyrus bretschneideri* Rehd.; should be an ecotype of *P. pyrifolia*)^25, 26^. The percentages of mapped reads were similar among the 21 libraries (72.75–77.32%), and 35.04–50.29% of the reads were perfectly mapped (Table 1 and Table S1).

**Table 1:** Statistics on the quality and output of the RNA-Seq libraries.

Classification	Maximum	Minimum	Average
No.raw reads	24,116,813	20,373,330	22,348,931
No.clean reads	23,864,720	19,711,240	21,544,238
No.mapped reads	18,227,873	14,339,927	16,141,316
% of mapped reads	77.32%	72.75%	74.87%
No.perfect mapped reads	12,231,683	8,349,296	9,746,449
% of perfect mapped reads	50.29%	35.04%	37.40%
Q30%	81.86%	80.06%	80.68%
Total base pair	91,377,116,282		
Coverage of the pear transcriptome	793×		

**Figure 2:**
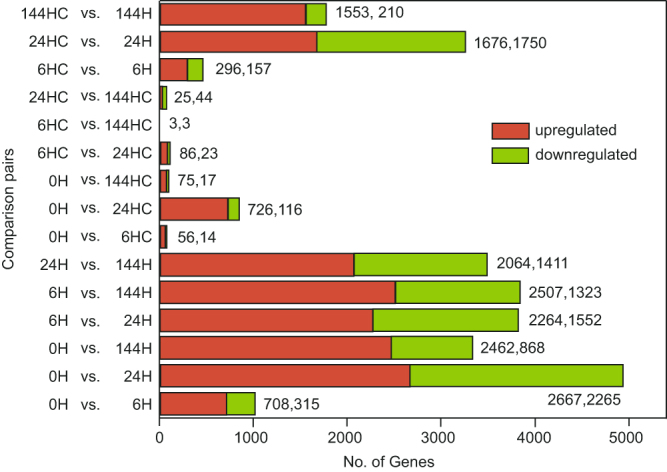
Comparison of the differential expressed genes of each pairs. The labels of the samples are displayed as “hours after bag-removal” (H) or “hours without bag-removal” (HC) Note that bag removal significantly changes the gene expression pattern in the fruit peel, while genes of the control had an expression pattern similar to the 0 h sample.

The total clean reads were assembled into transcripts and compared with the reference gene model (including 42,812 genes). In total, 31,581 known genes (73.57% of the total model genes) and 4,616 new transcripts were identified. After filtering out new transcripts shorter than 150 bp and those with only one exon, 1,198 transcripts were predicted as new genes. To obtain comprehensive information on the detected transcripts, both known and newly predicted genes were annotated based on five public protein databases. Differentially expressed genes (DEGs) were identified in each pair of samples using the criteria listed in the Material and Methods section. The genes with reads per kilobase of transcript per million mapped reads (RPKM) value in more than 12 samples lower than 0.3 were filtered out. Finally, 8,870 DEGs remained for further analysis (Fig. 2, Table S2).

### Validation of DEGs identified using RNA-Seq

Eight DEGs were randomly selected to have their transcript levels measured by qPCR. Although the exact fold change of the DEGs at several data points varied between RNA-Seq and qPCR, the differential expression trends detected by the two approaches were largely consistent (Fig. S1), indicating the reliability of the RNA-Seq results.

### Co-expression network analysis identified anthocyanin-related DEGs

**Figure 3:**
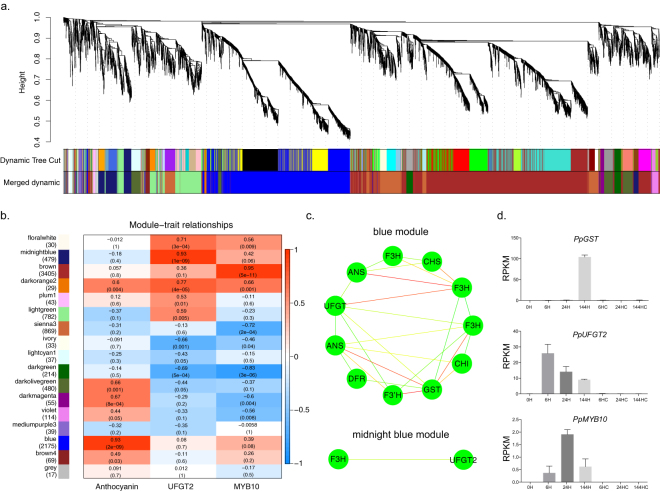
Weighted gene co-expression network analysis (WGCNA) of DEGs identified from ‘Meirensu’ pear peel after bag removal. (**a**) Hierarchical cluster tree showing 17 modules of co-expressed genes. Each of the 8,870 DEGs is represented by a tree leaf and each of the modules by a major tree branch. The lower panel shows modules in designated colours. (**b**) Module–trait correlations and corresponding *p*-values (in parentheses). The left panel shows the 17 modules and the number of member genes. The colour scale on the right shows module–trait correlations from −1 (blue) to 1 (red). The left panel “Anthocyanin” represents anthocyanin biosynthesis as a trait. The middle panel “UFGT2” represents the expression changes of *Pp*﻿*UFGT2*, which encodes the enzyme that catalyses the last step in anthocyanin biosynthesis, as a trait. The right panel “MYB10” represents the expression changes of *PpMYB10*, which is the key transcriptional factor activating anthocyanin biosynthesis, as a trait. (**c**) Cytoscape representation of co-expressed genes with edge weights ≥0.40 in module ‘blue’ and ‘midnight blue’. (**d**) RNA-Seq expression patterns of *Pp*﻿*GST*, *Pp* *UFGT2* and *Pp*﻿*MYB10*.

**Figure 4:**
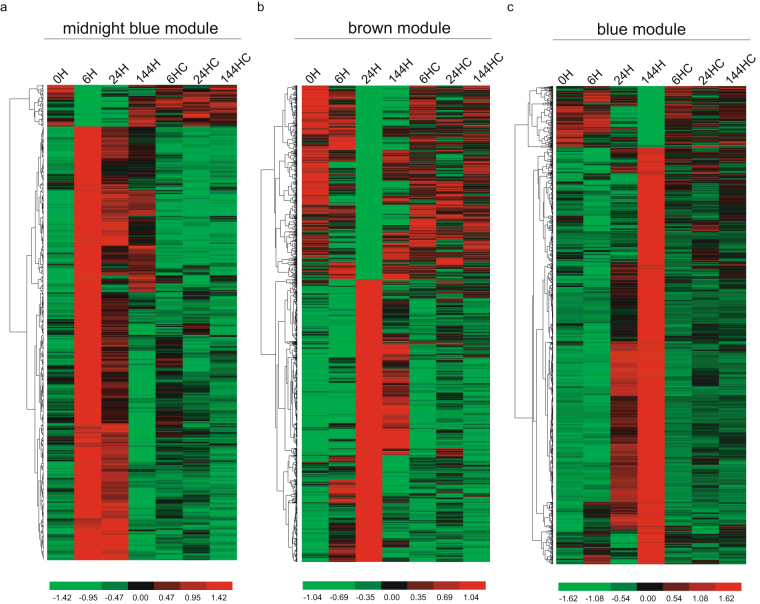
Heat maps showing the expression patterns of modules from the light-responsive genes.

Anthocyanin started to accumulate 72 h after bag removal (Fig. 1). To identify genes related to anthocyanin biosynthesis, we performed a weighted gene co-expression network analysis (WGCNA) using non-redundant DEGs. The analysis identified 13 WGCNA modules (Fig. 3a,b). An analysis of the module-trait relationships revealed that the “blue” module was highly correlated with the total anthocyanin content (r = 0.93, *p* = 2 × 10^−9^) in the 21 samples. In this module, we found 11 structural genes involved in anthocyanin biosynthesis and transportation. The expression patterns of these genes were highly connected even when we used a relatively stringent edge weight cut-off of 0.4 (Fig. 3c). Specifically, the *Glutathione S-transferase(GST)* gene had the greatest number of edges connecting to other genes, followed by a *UFGT* and an *F3H*. In addition, two structural genes, an *F3H* and an *UFGT2*, fell into the module of “midnight blue”. Interestingly, in our dataset, the main regulatory TF *PpMYB10* did not belong to these modules but to the “brown” module. A further module-trait relationship analysis, using the RPKMs of *P﻿p* *UFGT2* and *Pp﻿* *MYB10* as the trait data, revealed that their expression patterns were indeed highly related to the modules “midnight blue” and “brown”, respectively. We further revealed the expression patterns represented in these three modules (Fig. 3d). Genes belonging to module “blue” had an expression peak 144 h after bag removal, similar to the anthocyanin accumulation pattern, while genes in module “midnight blue” were highly expressed from 6 h after bag removal and then gradually declined. The expression pattern of the “brown” module, containing *PpMYB10*, had an expression peak at 24 h after bag removal (Fig. 4). We focused on these three modules in our further analysis.

Compared with the control samples, including the 0 h sample, the expression levels of the genes belonging to the “midnight blue” module significantly changed by 6 h after bag removal, and were considered early light-responsive genes. The genes in the “brown” module were considered middle light-responsive genes, while the genes in the “blue” module were late light-responsive genes.

### Functional annotations of light-responsive genes

**Figure 5:**
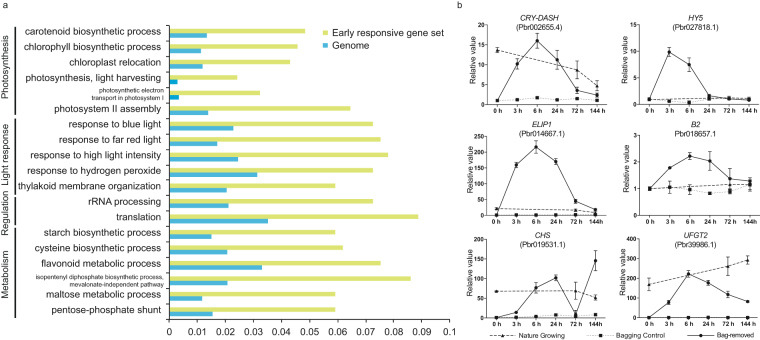
Analysis of the early-responsive genes. (**a**) Gene ontology enrichment analysis of early-responsive genes. (**b**) qPCR confirmation of the expression patterns of selected genes.

A Gene Ontology (GO) enrichment analysis of the early light-responsive genes identified 19 significantly enriched GO terms. Most of them were related to photosynthesis and light response, which are the typical light-induced pathways (Fig. 5, Table S3). In addition, some metabolism terms were also enriched (Fig. 5, Table S3). To obtain more detailed information, a pathway analysis was carried out using MapMan.

Based on the MapMan analysis, some primary metabolism-related pathway genes, including those involved in photosynthesis, light reaction, lipid biosynthesis and cell wall degradation, were in this gene set. However, genes belonging to carotenoid, flavonoid and lignin biosynthesis pathways were also observed in this gene set.

Because light was the most important environmental change after bag removal, a gene annotated as *CRY-DASH* and seven light-responsible genes, three *EARLY LIGHT-INDUCIBLE PROTEIN* genes, three *SUPPRESSOR PHYA-105* genes, and one light-inducible phototropism gene, were identified in the fruit’s early light-responsive gene set. However, other light photoreceptors were not found. In addition, some TFs that are involved in light signal transduction, including two *HY5* homologs and one *CONSTANS-like* (*COL*) B-box gene, were identified in this gene set (Fig. 5b).

The GO enrichment analyses of the middle and late light-responsive genes revealed distinct enrichment patterns, although both groups contained over 2,000 genes. In the middle light-responsive gene set, most of the enriched terms belonged to “cellular biosynthetic process”, including “pyrimidine ribonucleotide biosynthetic process” and “RNA methylation”. The enrichment of terms related to photosynthesis and light response pathways was not found at this stage (Table S3).

The functional annotation of the late light-responsive genes revealed that the most highly enriched GO terms were “defense response”, “response to chitin” and “immune response”. Most of these terms were involved in the responsive pathways. In the “metabolic process” category, “flavonoid biosynthesis”, “ethylene biosynthesis process”, “jasmonic acid metabolic process” and “cGMP biosynthetic process” were overrepresented. In addition, in the “biological regulation” category, “negative regulation of programmed cell death”, “regulation of hydrogen peroxide metabolic process” and “negative regulation of defense response” were highly enriched (Table S3).

### Genes involved in the biosynthesis and signal transduction of plant hormones

To determine the functions of plant hormones in the bagging-induced colouration, the expression patterns of genes involved in their biosynthesis and signal transduction were analysed. Significant expression changes were mostly observed in the middle- or late-light responsive stages, but a few genes, such as a *1-amino-cyclopropane-1-carboxylate synthase* (*PpACS)*, responded to the light condition change at the early stage. In addition, several genes related to the signal transduction of phytohormones were identified, and these were involved in auxin, ethylene, jasmonic acid (JA), abscisic acid (ABA), gibberellic acid (GA), brassinosteroid (BR) and cytokinin signalling pathways (Table 2).

**Table 2:** Selected differentially expressed genes related to the phytohormone biosynthesis and signalling pathways during light-responsive reactions.

Gene ID	Pathway	Module	Peak orientation	Annotation
Pbr041497.1	ABA	midnight blue	Up	protein phosphatase 2C 77-like isoformX1
Pbr026127.1	ABA	midnight blue	Up	magnesium-chelatase subunit ChlH, chloroplastic-like
Pbr019415.1	ABA	brown	Up	abscisic acid receptor PYL4-like
Pbr010794.1	ABA	brown	Up	abscisic acid receptor PYL8-like
Pbr003860.1	ABA	brown	Up	abscisic acid 8′-hydroxylase 2
Pbr007589.1	ABA	brown	Down	protein ABSCISICACID-INSENSITIVE 5-like
Pbr006776.1	ABA	blue	Up	abscisic acid 8′-hydroxylase1-like
Pbr030688.1	Auxin	midnight blue	Up	probable indole-3-acetic acid-amido synthetase GH3.5
Pbr008163.2	Auxin	brown	Down	auxin transporter-like protein 3
Pbr013531.1	Auxin	brown	Up	auxin-induced protein 15A-like
Pbr004491.1	Auxin	brown	Up	indole-3-acetic acid-induced protein ARG7-like
Pbr022122.1	Auxin	brown	Down	auxin-induced protein15A
Pbr021158.1	Auxin	brown	Down	indole-3-acetic acid-amido synthetase GH3.6-like
Pbr000415.1	Auxin	brown	Down	auxin response factor 3- like
Pbr021934.1	BR	brown	Down	brassinosteroid LRR receptor kinase-like
Pbr021939.1	BR	brown	Down	brassinosteroid LRR receptor kinase-like
Pbr001823.1	BR	brown	Down	cytochrome P450 734A1
Pbr004288.1	BR	blue	Up	BRASSINOSTEROID INSENSITIVE1-associated receptor kinase 1-like
Pbr012231.1	BR	blue	Up	delta(24)-sterol reductase
Pbr023118.1	BR	blue	Up	receptor-like protein kinase BRI1-like3
Pbr011993.1	Cytokinin	brown	Down	two-component response regulator ARR1-like isoform X2
Pbr005849.1	Cytokinin	brown	Up	cytokinin riboside 5′-monophosphate phosphoribohydrolase LOG3-like
Pbr009698.1	Cytokinin	brown	Up	cytokinin dehydrogenase 6-like
Pbr015617.1	Cytokinin	brown	Up	cytokinin dehydrogenase 3-like
Pbr015575.1	Ethylene	midnight blue	Up	1-aminocyclopropane-1-carboxylate synthase 7
Pbr023044.1	Ethylene	brown	Up	ethylene-responsive transcription factor 1B-like
Pbr008360.1	Ethylene	brown	Down	ethylene-insensitive protein 2
Pbr005179.1	Ethylene	brown	Up	1-aminocyclopropane-1-carboxylate oxidase
Pbr011802.1	Ethylene	brown	Down	5′-3′ exoribonuclease 3-like isoform X1
Pbr004323.1	Ethylene	blue	Down	protein EIN4-like
Pbr004403.1	Ethylene	blue	Up	5′-3′ exoribonuclease 4 isoform X1
Pbr015589.1	Ethylene	blue	Up	1-aminocyclopropane-1-carboxylate oxidase 1
Pbr015895.1	Ethylene	blue	Down	probable amino transferase ACS10
Pbr036605.1	GA	brown	Down	transcription factor TGA2-like
Pbr017104.1	GA	brown	Down	gibberellin receptor GID1C-like
Pbr013018.1	GA	brown	Down	gibberellin 2-beta-dioxygenase 8-like
Pbr014064.1	GA	brown	Down	DELLA protein GAI-like
Pbr039229.1	JA	brown	Up	protein TIFY10B-like
Pbr003675.1	JA	blue	Up	protein TIFY9-like
Pbr027730.1	JA	blue	Up	protein TIFY10A
Pbr011711.1	JA	blue	Up	protein TIFY3B-like
Pbr037418.1	JA	blue	Up	protein TIFY10A-like

### Transcription factors of light-responsive genes

In the early light-responsive gene set, only 10 TFs were identified. In addition to the two *HY5*s and one *COL* mentioned above, one *bHLH*, two *C2H2*, two *CCAAT* box-binding factors, one *MYB* gene and one *WRKY* gene were identified. Interestingly, unlike other TFs that were upregulated at 6 h, the expression levels of *MYB* and *WRKY* were down-regulated, suggesting that they had distinct regulatory functions during the light-response process.

**Figure 6:**
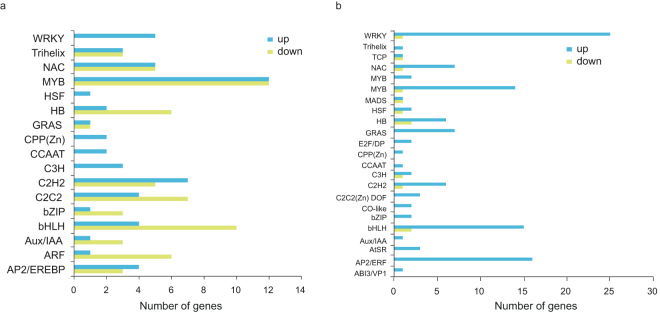
Transcription factor analysis of middle- (**a**) and late- (**b**) responsive genes. The numbers of up-regulated genes and down-regulated genes comparing to 0 h were showed with blue (up) and green (down), respectively.

In the middle stage, 134 genes were assigned to the MapMan “transcription factors” bin and more than half were down-regulated. Members of the WRKY, CCAAT, C3H, HSF and CPP families were upregulated, while other TF families were regulated in both directions. Of these TFs, MYB was the largest family with 24 members, including *PpMYB10*, which is the key regulator of anthocyanin biosynthesis (Fig. 6a).

Although there were similar numbers of TFs in the late stage as in the middle stage, most of them were upregulated. In this gene set, WRKY was the largest family with 26 members, followed by the bHLH, AP2/ERF and MYB families, with 17, 16 and 15 members, respectively (Fig. 6b).

### Various expression patterns of anthocyanin biosynthesis structural genes

**Figure 7:**
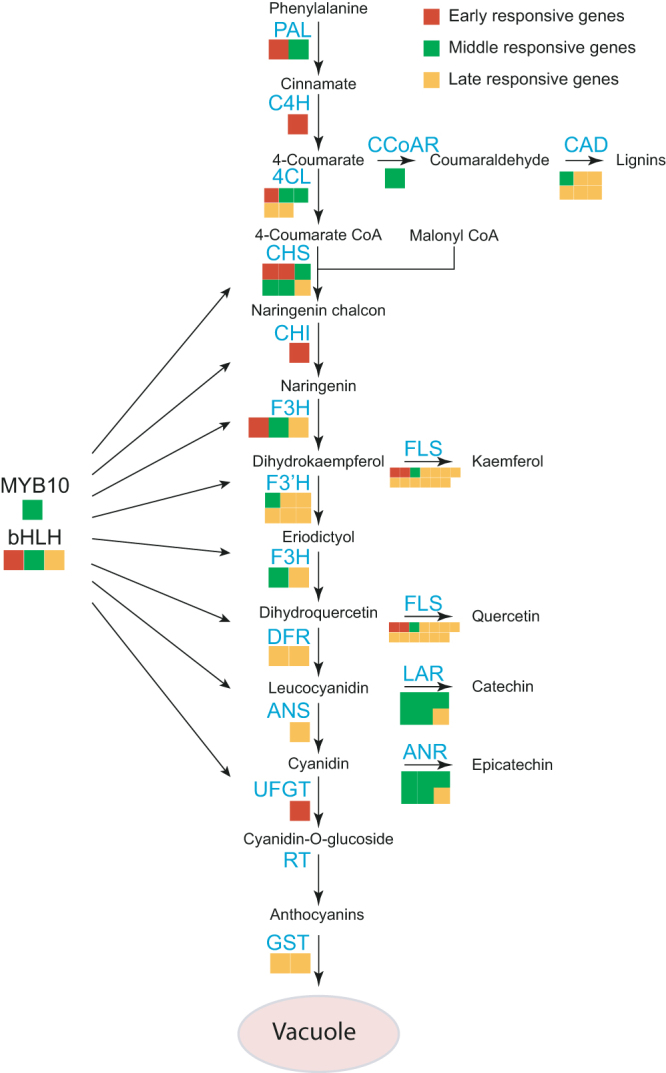
Categorisation of genes belonging to the phenylalanine pathway. Structural genes within the phenylalanine pathway were assigned to different light-responsive gene sets.

Because the biosynthesis of anthocyanin is catalysed by multiple enzymes that are encoded by structural genes in the phenylpropanoid pathway (Fig. 7), we mapped the light-responsive genes to this pathway to identify the expression patterns of the anthocyanin biosynthesis-related genes. The genes in the early biosynthesis stage, such as *PpCHS*s and *PpCHI*s, were upregulated relatively early, while other genes, such as *PpF3H*s, *PpF3′H*s, *PpDFR*s and *PpANS*s, were upregulated later. Interestingly, *PpUFGT2*, the last enzyme in anthocyanin biosynthesis, was upregulated 6 h after bag removal. The key regulatory gene *PpMYB10*, together with some *PpCHS*s, *PpF3H*s and *PpF3*′*Hs*, peaked at 24 h after bag-removal, which was prior to the anthocyanin accumulation. In addition, the members of the bHLH family had different expression patterns, as shown in Fig. 6.

**Figure 8:**
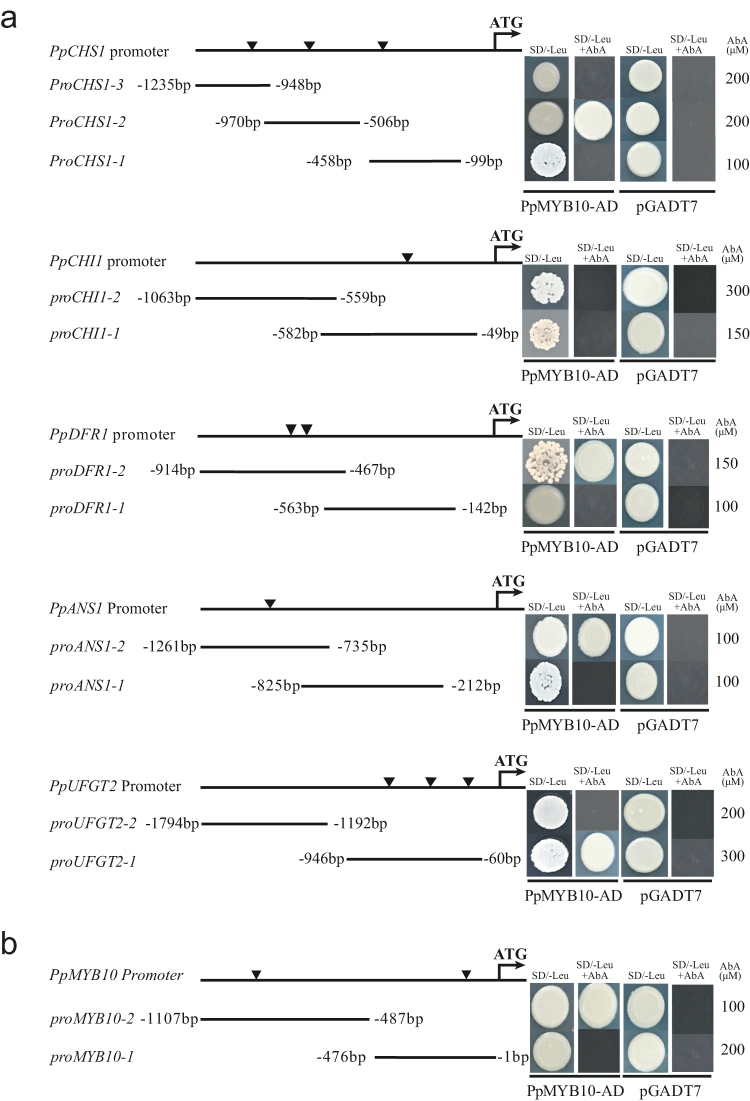
Direct binding of PpMYB10 on the promoter of anthocyanin biosynthetic structural genes (**a**) and *PpMYB10* itself (**b**) of ‘Meirensu’ in yeast-one hybrid system. The tri-angle marked the conserved MYB binding domain predicted using PlantCare.

Previous reports showed that most anthocyanin biosynthetic genes were regulated by MYB family, potentially PpMYB10 in pear. As *PpMYB10* is the middle light-responsive gene while some anthocyanin biosynthetic genes were late-responsive genes, their direct interaction were further tested in yeast-one hybrid system. PpMYB10 directly bound the promoters of *PpCHS1* (Pbr019531.1), *PpDFR1* (Pbr038148.1), *PpANS1* (Pbr001543.2) and *PpUFGT2* (Pbr039986.1) in yeast-one hybrid system (Fig. 8a) while the interaction between PpMYB10 and *PpCHI* promoter were not detected. In all these binding events, the presence of conserved MYB-binding motif were identified. In addition, PpMYB10 also bound its own promoter (Fig. 8b), suggesting a self-enhanced regulatory pathway during the pear fruit colouration.

## Discussion

The responses to bagging treatment and postharvest irradiation in the RCSP are distinct from those of European pears. During post-harvest irradiation after bag removal, RCSPs gradually accumulate anthocyanin in the fruit skin, while European pears show almost no change^1, 2, 27^. In addition, the accumulation of anthocyanin in the RCSPs accompanies the maturation process, whereas European pears lose some of the red colour as the harvest time approaches, indicating that different triggers mediate the red colouration in these two species. In apple, bagging-induced colouration is mainly mediated by light and enhanced by a relatively low ambient temperature^7, 28^. This probably involves light-responsive genes, such as *HY5* and *COP1*, and the flavonoid biosynthesis pathway^22, 23^. In our results, the red colour of ‘Meirensu’ only developed in the fruits that were removed from bags, confirming that the red colouration of RCSP is a light-dependent process, similar to the colouration of apple.

The bagging, followed by bag removal, is a transition from long-term darkness to light, which triggers a response to oxidative stress in the fruit peel that includes the accumulation of lignin and flavonoids, in addition to the scavenging enzymes of reactive oxygen species^29^. Based on the gene expression patterns during this process, we identified three responsive stages to light in pear fruit peels. In the early-responsive stage, GO terms related to light response and photosynthesis were highly enriched (Fig. 5). In the middle responsive stage, most of these GO terms were not enriched, while the “cellular biosynthetic process” was highly enriched. In the late responsive stage, “flavonoid biosynthesis”, “hormone metabolism” and “defense response” GO terms were overrepresented (Table S﻿3). These results indicated a sequential light-responsive reaction in fruit peel. However, whether these pathways contribute to anthocyanin biosynthesis needs further investigation.

### Upstream *trans*-regulator of *PpMYB10*

MYB10 is the critical light-inducible TF for anthocyanin biosynthesis^7–9, 30^. However, how *MYB10* is transcriptionally regulated is still not clear, except that it involves *HY5* and the B-box gene *MdCOL11* ^22, 28^. In our dataset, *PpMYB10* was categorised in the middle light-responsive gene set that had peaked expression levels 24 h after bag removal. As high expression levels of *HY5* and *COL5*, which are both light-related genes, were observed in the early-responsive stage, it is reasonable to speculate that both of these genes directly, or indirectly, induced the expression of *PpMYB10*, which further triggered the expression of downstream structural genes.

In *Arabidopsis*, HY5 is a pivotal TF for photomorphogenesis that is regulated by multiple photoreceptors and the COP1 protein degradation machinery^31–33^, and directly binds to the G-box in the promoter of *AtMYB75*, the Arabidopsis homolog of *PpMYB10*, to induce its expression^34^. In apple, MdHY5 also bound on the 5′ upstream region of *MdMYBA*, at least in a yeast system^22^, although the direct activation effects were still not observed. The high expression of *PpHY5* immediately after bag removal is in accordance with our expectations. However, more studies are needed to characterize whether there is direct interaction between PpHY5 and *PpMYB10* to unveil the regulatory pathway and further explain the light-induced anthocyanin biosynthesis.

The CO-like family, also known as the BBX family, contains an N-terminal zinc-binding B-box motif and functions as a transcriptional regulator in response to light, circadian cues and brassinosteroid-light crosstalk signalling. Some members of the COL family have been characterized as positive regulators of anthocyanin biosynthesis. The overexpression of apple *MdCOL11* in *Arabidopsis* increased anthocyanin biosynthesis and promoted earlier flowering under long-day conditions^28^. PpCOL5 is also a member of the COL family. In *Arabidopsis*, AtCOL5, the homolog of PpCOL5, functions in controlling the flowering time under short-day conditions in a CO-independent pathway^35^. In our dataset, *PpCOL5* was significantly upregulated immediately after bag removal (Fig. 5), suggesting it has a potential role in the light-responsive pathway. However, owing to limited reports on the *COL5* gene in model plants, we are unable to conclude that PpCOL5 is involved in the regulation of anthocyanin biosynthesis. However, because MdCOL11 can simultaneously induce biosynthesis and flowering, PpCOL5 may also participate in light-induced anthocyanin biosynthesis. In addition, because HY5 lacks the *trans*-activation domain and is, therefore, unable to activate transcription, at least in yeast^36^ and in plant cells^37^, its *trans*-activity must require a cofactor^31, 37^. Although BBX21 and BBX22 have been proposed as cofactors for HY5, no solid evidence has been provided^38, 39^. The similar expression patterns of *PpHY5* and *PpCOL5* suggest that COL5 may be another candidate cofactor of HY5.

### Other TFs related to anthocyanin biosynthesis

The differential expression patterns of many other TFs, especially some *MADS-box*, *NAC* and *MYB* genes, were recorded in our datasets, suggesting that they had potential roles in anthocyanin biosynthesis. *MADS-box* genes were also involved in the anthocyanin biosynthesis in European pear in a MYB-independent manner, especially during early development^40^, but their functions in the light-responsive process still needs further studies. NAC also participates in the modulation of anthocyanin biosynthesis by directly regulating *MYB10* genes in peach^41^. Here, although we identified many light-induced or -suppressed TFs, their functions in anthocyanin biosynthesis need further investigation.

### Phytohormones are involved in bagging-induced fruit colouration

Applying exogenous hormones affects the light-dependent accumulation of anthocyanin and the signalling pathways triggered by plant hormones interact with those triggered by light^42^. We also identified genes, several enriched GO terms, and DEGs related to phytohormones mostly during the late-responsive stage (Table 2). Although we cannot conclude that the differentially expressed hormone-related genes contributed to anthocyanin biosynthesis, it is reasonable to speculate that some phytohormones indeed affected the bagging-induced anthocyanin biosynthesis.

Exogenous methyl jasmonate (MeJA) can induce the fruit colouration in apple and pear^3, 43, 44^. Coronatine insensitive1 (COI), an F-box protein that forms a complex with ASK1/ASK2, Cullin1 and Rbx1, is the key player in diverse JA responses^45^. In its *Arabidopsis* mutant, *coi-1*, anthocyanin fails to accumulate in response to MeJA owing to the reduced expression of three anthocyanin regulatory factors, *MYB75*, *MYB90* and *GLABRA 3* ^46–48^. In apple, the mechanism by which MeJA induces the anthocyanin accumulation is also well characterised^44^. The apple jasmonate zim domain (JAZ) protein interacts with bHLH3 to attenuate the formation of an MdMYB9-involved MBW complex that promotes the transcription of structural genes. Upon the perception of a JA signal, the JAZ protein is degraded through the COI-mediated 26S proteosome pathway, releasing bHLH for the formation of MBW, which activates the downstream structural genes^44^. In our results, we found six differentially expressed JAZ genes (Table 2), most of which were upregulated in the middle- or late-responsive stages, opposite to our expectations. Two possible explanations for this result are as follows: 1) Because JAZs are also subjected to post-translational regulation, the increased transcription level did not equal the high level of translated protein^48^, or 2) The JA-induced and light-induced anthocyanin biosyntheses activated different MYB proteins^44^, and the increase of JAZ did not affect the expression and function of *PpMYB10*, which is the essential light-inducible anthocyanin-related MYB protein.

The effects of ethylene on the anthocyanin accumulation vary among species. As ethylene biosynthesis and anthocyanin accumulation are coherently regulated during fruit ripening, it is thought that ethylene is a positive regulator of anthocyanin biosynthesis. The treatment of grape berries with 2-chloroethylphosphonic acid activated the transcription of structural genes encoding the key enzymes of anthocyanin biosynthesis and increased anthocyanin accumulation^49^. A similar phenomenon was also observed in apple^50^. However, ethylene plays a negative regulatory role in anthocyanin biosynthesis in some other species, such as red cabbage, tobacco and corn^51–54^. Specifically, sugar-induced anthocyanin accumulation is suppressed by ethylene in the model plant *Arabidopsis*. Ethylene suppresses anthocyanin accumulation by binding to redundant receptors, such as ETHYLENE RESPONSE 1 (ETR1), ETR2, ETHYLENE RESPONSE SENSOR 1 (ERS1) and ERS2, with ETR1 possibly playing a dominant regulatory role^55^. Ethylene tends to enhance the nuclear localisation of COP1, thus inhibiting the activity of HY5 and promoting hypocotyl growth^56^ under light conditions or seed germination under salt stress conditions^57^. The antagonistic regulation between ethylene and light on COP1-HY5 pathway is essential for anthocyanin biosynthesis, suggesting that ethylene may suppress anthocyanin biosynthesis through the COP1-HY5 pathway. However, we observed that the anthocyanin accumulation, along with the increased transcription of *PpHY5* and *PpACS*s (Fig. 4, Table 2), suggested that the anthocyanin accumulation in the pear peel may be subjected to complex regulation and that ethylene may have different functions in the regulation of anthocyanin biosynthesis through various pathways.

GA suppresses anthocyanin biosynthesis through the DELLA proteins^58–60^. DELLA proteins are negative regulators of GA signalling and positively regulate anthocyanin biosynthesis. In DELLA protein quadruple mutants (*gai-t6*/*rga-t2*/*rgl1-1*/*rgl2-1*), the expression of anthocyanin biosynthesis genes, such as *ANS* and *F3′H*, decreased, while the expression of *UFGT* and *PAP1* is independent of the DELLA proteins^58^. The DELLA-promoted anthocyanin biosynthesis is partially mediated by tMYBL2, an inhibitor of transcription, and JAZ regulatory proteins^60^. In this sense, GA-mediated anthocyanin inhibition is at least partially independent of the light-induced anthocyanin biosynthesis pathway in which PAP1 was involved. However, GA is also involved in the HY5-mediated skotomorphogenesis by reducing the abundance of the HY5 protein through the modulation of COP1 activity^61^, potentially indirectly regulating anthocyanin biosynthesis. In the present study, we observed the downregulation of two DELLA proteins, suggesting that GA was also involved in the light-induced anthocyanin biosynthesis in pear peel. However, how GA is involved in the fine-tuned regulation of light-induced anthocyanin biosynthesis needs to be studied further.

### Differential regulation of structural genes for anthocyanin biosynthesis

Several reports proposed that both the early and late anthocyanin biosynthetic genes could be activated by MYB10^7, 8^. However, from the temporal expression patterns of these genes, it appears that these genes are differentially regulated, which were further confirmed by yeast-one hybrid assay. Some lines of evidence show that the expression of *UFGT* is coupled with that of *MYB10* in other plants, while the expression of other genes are not^30, 62^. In our work, the early-stage biosynthetic genes, such as *PpCHS1* and *PpCHI1*, are upregulated immediately after bag removal (Fig. 7), which is much earlier than the anthocyanin accumulation and the high expression of *PpMYB10*, but PpMYB10 is capable to bind the promoters of *PpCHS1* but not *PpCHI1* (Fig. 8). These results suggested that PpMYB10 directly regulate *PpCHS1* but other regulatory were also involved in. Indeed, HY5 directly activates *AtCHS* by binding to the G-box motif in the *Arabidopsis* promoter^36, 63^, suggesting a function for PpHY5 in the light-induced flavonoid biosynthesis in RCSP. On the other hand, some of the late-stage biosynthetic genes, such as *PpDFR* and *PpANS*, were induced in the late-responsive stage, along with the accumulation of anthocyanin, are mainly under the regulation of PpMYB10 (Fig. 8). In our dataset, the expression of *PpUFGT2* peaked 6 h after bag removal, much earlier than *PpMYB10*, which was further confirmed by qRT-PCR, indicating a novel regulation of *PpUFGT2* in RCSP ‘Meirensu’, although it is still under the regulation of PpMYB10. Because the expression pattern of *PpUFGT2* is similar to that of *HY5*, and a G-box motif is found in the 5′ upstream region of *PpUFGT2*, HY5 may directly activate the expression of *PpUFGT2* by binding the G-box. Interestingly, we observed the direct binding of PpMYB10 on the promoter of itself, which may explain the later appearance of the peak than *PpHY5*.

**Figure 9:**
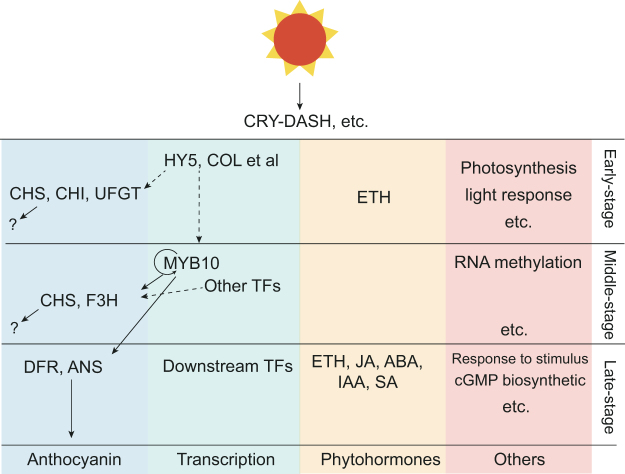
Putative model for bag removal-induced anthocyanin biosynthesis in ‘Meirensu’ pear peels. The phytohormone and other pathways based on the gene ontology enrichment analysis.

In summary, a transcriptional regulatory model (Fig. 9) showing the light-responsive reactions in pear fruit peel was inferred from the transcriptome analysis. After bag removal, photoreceptors, such as CRY-dash, received the light, triggering the light-responsive pathway, activating the transcription of *HY5* and *COL5*, and inducing the transcription of downstream TFs, such as *MYB10*, and structural genes, *CHS*, *F3H* and *UFGT2*, which resulted in the accumulation of anthocyanin in the fruit peel. In addition, plant hormone- and other biological process-related pathways were activated by the light-responsive pathway, resulting in significant physiological changes in fruit after bag removal, including anthocyanin accumulation in the fruit peel.

Anthocyanin biosynthesis is a complex process involving various regulatory pathways. Our work provided a transcriptomic view on the light-induced anthocyanin biosynthesis that occurs after bagging treatment. However, owing to technical limitations, our work did not provide any post-transcriptional or (post)-translational evidence for anthocyanin biosynthesis, which are also importation regulatory pathways for red pigmentation^64, 65^. Whether these pathways are also involved in the bagging-improved colouration needs further analysis.

## Materials and Methods

### Plant materials and experimental treatments

The fruit of RCSP ‘Meirensu’ were obtained from a commercial orchard in Kunming, Yunnan Province, China. In total, 500 fruitlets were covered with double layers of yellow-black paper bags [Kobayashi (Qingdao) Co., Ltd., Qingdao China] 40 days after full blossom (DAFB). Half of the fruits had their bags removed 10 days before harvest (155 DAFB), and the others remained bagged as controls. 30 fruits were randomly sampled at 0, 3, 6, 24, 72 and 144 h after bag removal and divided to three biological replicates. Control fruit were sampled at the same times and named as 3 h-C, 6 h-C, 24 h-C, 72 h-C and 144 h-C (C, control; the 0 H sample did not require a control). Fruit peel was scraped and immediately frozen in liquid nitrogen, and stored at −80 °C until use.

### Measurements of total anthocyanin, chlorophyll and carotenoid concentrations

The anthocyanin concentration was measured according to a methanol-HCL method and was presented as mg cyanidin-3-galactoside per 100 g fresh tissue. One gram of fruit peel was mixed with methanol containing 0.1% HCl, followed by centrifugation at 4 °C and 18,000 *×* *g* for 20 min. The absorbance of each 100-μL extract was assessed using a spectrophotometer (DU800; Beckman, IN, USA) at 510 and 700 nm, in buffers of pH 1.0 and 4.5, respectively. The anthocyanin concentration was calculated using the equation: A = [(A510–A700)_pH1.0_ − (A510–A700)_pH4.5_] with a molar extinction coefficient of 3.02 × 10^4^ cyanidin-3-galactoside^66^. One gram of ground fruit peel was homogenized in 6 mL of 80% cold acetone and centrifuged at 4 °C and 12,000 rpm for 20 min. The extract’s absorbance was measured using a spectrophotometer (DU800; Beckman) at 440, 645 and 663 nm. The chlorophyll concentration was calculated using the equation Ct = 20.2 A645 + 8.02 A663, and the carotenoid concentration was calculated based on the equation Ck = 4.7 A440 − 0.27 Ct^67^.

### RNA extraction, library preparation and RNA-Seq

Total RNA from each sample was isolated three times, as biological replications, using a modified cetyltrimethylammonium bromide method^68^. The RNA quality was assessed using an Agilent 2100 Bioanalyzer (Santa Clara, CA, USA). Only the samples with RNA integrity numbers >7.5 were used for deep sequencing. mRNA was enriched and purified, and then cleaved into small pieces. Short fragments were purified with a QiaQuick PCR extraction kit (Qiagen, Venlo, Netherlands) and dissolved with the EB buffer supplied in the kit for end preparation and poly(A) addition. Then, the short fragments were connected with sequencing adapters. After agarose gel electrophoresis, suitable fragments were selected as templates for PCR amplification, and the library was sequenced using an Illumina HiSeq™2000 (San Diego, CA, USA) by Biomarker (Beijing, China).

### Sequence data processing and mapping reads to the pear genome

To obtain high-quality clean read data for sequence analysis, adaptor sequences, empty reads, low-quality sequences with greater than 5% N (the percentage of nucleotides in the reads that could not be sequenced) and those containing more than 10% ambiguous bases were removed. Clean reads were mapped to the pear genome sequence (http://peargenome.njau.edu.cn/) using TopHat^69^ with default parameters. The reads were then assembled into transcripts and compared with reference gene models using Cufflinks^69^. Transcripts that did not exist in the CDS database of the pear genome were extracted to predict new genes using the EMBOSS package (http://emboss.open-bio.org/).

### Functional annotations of known and new genes

All of the known and newly predicted genes were annotated with protein databases, including the NCBI nonredundant, Swiss-Prot, Kyoto Encyclopedia of Genes and Genomes and Cluster of Orthologous Groups databases, using the BLASTX algorithm with a cutoff e-value of 10^−5^. Blast2GO (http://www.blast2go.com) was used to obtain GO annotation of the transcripts. The GO enrichment analysis of DEGs was implemented by the Cytoscape plugin BinGO^70^. GO terms with corrected *p*-values < 0.05 were considered significantly enriched.

### Identification of significant DEGs

A statistical analysis of the frequency of each gene in each library was performed to compare gene expression levels. All of the clean reads were aligned to genes using SOAP aligner^71^ and then normalized into RPKM values^72^. Transcript abundance differences between each pair of samples were then calculated based on the ratios of the RPKM values, and the false discovery rate control method was used to identify the *p*-value thresholds in multiple tests to compute the significance of any differences in transcript abundance^73^. Genes with an absolute log_2_ratio ≥ 2 and a false discovery rate significance score < 0.001 were considered significantly differentially expressed.

### Construction and visualization of co-expression network

The DEGs were then used to perform the co-expression network analysis using the R package WGCNA^74^. The co-expression network was visualized using the free software Cytoscape^75^. The expression data were clustered with Cluster 3.0^76^ and displayed using Java Treeview^77^.

### Real-time quantitative RT-PCR (qPCR) analysis

Total RNAs were reverse transcribed to cDNAs using an RTase (Takara Bio, Kusatsu, Japan), following the manufacturer’s instructions, and subjected to qPCR as described in Zhang, *et al*.^27^. Primers used for qRT-PCR was listed in Table S4.

### Yeast One-hybrid assay

The yeast one-hybrid assays were performed using the Matchmaker Gold Yeast One-Hybrid System Kit (Clontech) according to the manufacturer’s instruction. The primers used for construction were listed in Table S4.

### Statistical analysis

*t*-test (*p* = 0.05) were performed using the Data Processing System (DPS, version 3.01; Zhejiang University, Hangzhou, China).

### Data accessibility

The sequencing data have been deposited in the NCBI Sequence Read Archive (http://www.ncbi.nlm.nih.gov/sra/), with sample accession numbers SAMN03857509, SAMN03857510, SAMN03857511, SAMN03857512, SAMN03857513, SAMN03857514 and SAMN03857515.

## Electronic supplementary material

Table S1

Table S2

Table S3

Table S4

Supplementary_figures
