# Supplementary material for: Transcriptome analysis of bagging-treated red Chinese sand pear peels reveals light-responsive pathway functions in anthocyanin accumulation
Source: Sci Rep. 2017 Mar 3;7:63. doi: 10.1038/s41598-017-00069-z (PMC5428347; doi:10.1038/s41598-017-00069-z)
Supplement: Supplementary file 5 — Supplementary_figures [file 41598_2017_69_MOESM5_ESM.doc]

**Supplementary Fig.**

**Transcriptome analysis of bagging-treated red Chinese sand pear peels reveals light-responsive pathway functions in anthocyanin accumulation**

**Songling Bai^1,3,4,#^, Yongwang Sun^1,3,4,#^, Minjie Qian^1,3,4^, Fengxia Yang^1,3,4^, Junbei Ni^1,3,4^, Ruiyan Tao^1,3,4^, Lin Li^5^, Qun Shu^5^, Dong Zhang^2*^ and Yuanwen Teng^1,3,4*^**

# ^1^ Department of Horticulture, Zhejiang University, Hangzhou 310058, Zhejiang, PR China

# ^2^ College of Horticulture, Northwest A & F University, Yangling 712100, Shaanxi, PR China

# ^3^ The Key Laboratory of Horticultural Plant Growth, Development and Quality Improvement, the Ministry of Agriculture of China, Hangzhou 310058, Zhejiang, PR China

# ^4^ Zhejiang Provincial Key Laboratory of Integrative Biology of Horticultural Plants, Hangzhou 310058, Zhejiang, PR China

# ^5^ Institute of Horticulture, Yunnan Academy of Agricultural Sciences, Kunming 650205, Yunnan Province, PR China

# These authors equally contributed to this work.

**^*^** To whom correspondence should be addressed. Tel.: +86 571 88982803; fax: +86 571 88982803. E-mail address: ywteng@zju.edu.cn (Y. Teng), afant@nwsuaf.edu.cn (D. Zhang)


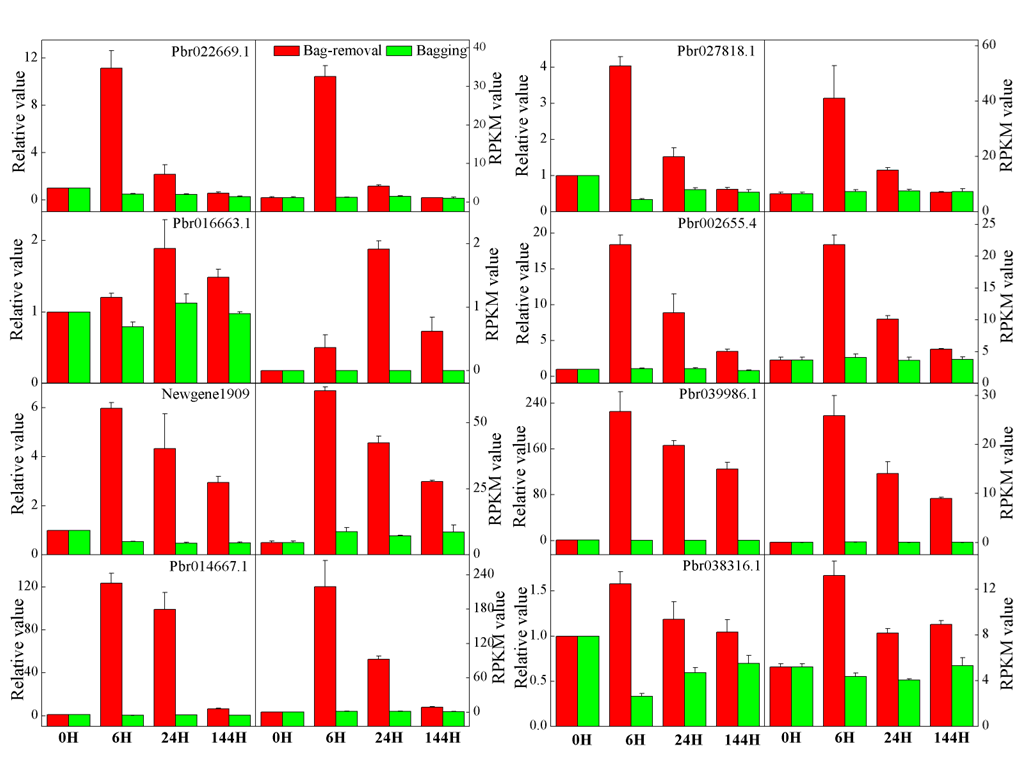


**Fig. S1. qPCR validation of differentially expressed genes (DEGs) in red Chinese sand pear peel detected by RNA-Seq.** The qPCR data (left) are shown with the RNA-Seq data (right).
